# Occult Hepatitis B: Clinical Viewpoint and Management

**DOI:** 10.1155/2013/259148

**Published:** 2013-03-04

**Authors:** Mehdi Zobeiri

**Affiliations:** Internal Medicine Department, Imam Reza Hospital, Kermanshah University of Medical Sciences, Kermanshah, Iran

## Abstract

Occult HBV infection (OBI) is defined as HBV DNA detection in serum or in the liver by sensitive diagnostic tests in HBsAg-negative patients with or without serologic markers of previous viral exposure. OBI seems to be higher among subjects at high risk for HBV infection and with liver disease. OBI can be both a source of virus contamination in blood and organ donations and the reservoir for full blown hepatitis after reactivation. HBV reactivation depends on viral and host factors but these associations have not been analyzed thoroughly. In OBI, it would be best to prevent HBV reactivation which inhibits the development of hepatitis and subsequent mortality. In diverse cases with insufficient data to recommend routine prophylaxis, early identification of virologic reactivation is essential to start antiviral therapy. For retrieving articles regarding OBI, various databases, including OVID, PubMed, Scopus, and ScienceDirect, were used.

## 1. Introduction

Hepatitis B virus (HBV) infection is a major global health problem with about 350–400 million chronically infected individuals [1]. HBV infection can induce a wide spectrum of clinical features, ranging from an inactive carrier state to fulminate hepatitis, cirrhosis, or hepatocellular carcinoma [2]. According to European Association for the Study of the Liver (EASL), about one-third of the world's populations have serological evidence of past or present HBV infection [3]. Many of these individuals may unknowingly carry the virus for several years after recovery from acute hepatitis B without showing any clinical or biochemical evidence of liver disease, and serological markers can identify different clinical states of viral persistence [4, 5]. HBV infection is usually diagnosed when circulating hepatitis B surface antigen (HBsAg) is detected. Chronic infection is characterized by persistence of this antigen and presence of HBV DNA in serum and resolved HBV infection when patients show seropositivity for –HBc and HBs antibodies.

Occult hepatitis B infection (OBI) is defined as the existence of low-level HBV DNA in the serum (<200 IU/mL), cells of the lymphatic (immune) system, and/or hepatic tissue in patients with serological markers of previous infection (anti-HBc and/or anti-HBs positive) and the absence of serum HBsAg. More than 20 percent of patients had no serologic markers because the antibody titer may become undetectable over time, leaving HBV DNA as the only marker of the infection. Thus depending on the HBV antibodies (anti-HBc and/or anti-HBs), OBI may be seropositive or seronegative [2, 3].

## 2. Diagnosis

The gold standard for diagnosis of OBI became possible by highly sensitive and specific molecular biology techniques like HBV nucleic acid amplification testing (NAT), a PCR technique with detection limits of <10 copies HBV DNA per reaction [3]. Samples for analysis include specimens from the liver and blood but diagnosis of OBI most commonly is based on the analysis of serum samples, because liver specimens are only available in a minority of cases and standardized assays for use in liver tissue are not yet available [3]. If highly sensitive HBV DNA testing is not feasible, anti-HBc should be used as a less than ideal surrogate marker for identifying potential seropositive OBI individuals in cases of blood, tissue, or organ donation, and when immune suppressive therapy has to be administered [6]. In this context, it has to be stressed that not all anti-HBc-positive individuals are found to be HBV DNA positive and that anti-HBc tests may provide false positive results [7].

OBI is more often detected in patients positive for anti- HBc but negative for anti-HBs, presumably because these patients lack the neutralizing effect of anti-HBs [8]. Most frequently, seropositive occult HBV infection follows resolution of acute hepatitis and continues indefinitely after clearance of HBsAg and biochemical improvement in liver function; it can also occur after years of chronic HBsAg-positive infection [9, 10]. OBI must be differentiated from S-escape mutants infection, in which undetectable HBsAg is present in spite of the episomal, free HBV genomes at intrahepatic level as in overt infection. These cases (so-called false occult HBV) result from HBV strains with key mutations in pre-S region which is not recognized by commercially available kits, even when the most sensitive ones are used [11]. 

False OBI has been reported in up to 40% of patients with OBI [3, 11–13]. In the HBsAg assays, the use of multivalent anti-HBs antibodies is recommended for detection of false OBI [3].

## 3. Pathogenesis of HBV Persistence

OBI is mostly due to the indefinitely intrahepatic persistence of the viral genome of wild-type HBV (without any mutations in the precore and core promoter regions) [14]. Strong suppression of viral replication and gene expression by antiviral cytotoxic T cells is responsible for the very low or undetectable levels of serum HBV DNA in OBI [15]. Conversion of viral genome to a covalently closed circular DNA (cccDNA), which is formed in the nuclei of infected hepatocytes within the first 24 h following virus inoculation and forming of a minichromosome after binding to proteins, is the molecular basis of persistence [11, 16].

The stability and long-term persistence of viral cccDNA molecules, together with the long half-life of hepatocytes, imply that HBV infection, once it has occurred, may possibly continue for life [17].

The cccDNA correlation with serum HBV DNA is poor, especially among HBeAg-negative individuals [18]. 

Clinical utility of intrahepatic cccDNA assay is very limited due to the invasiveness of liver biopsy but a more realistic approach is the quantitative measurement of serum HBsAg, which correlates well with intrahepatic cccDNA levels in both HBeAg-positive and -negative patients [18]. 

cccDNA is the main template for the transcription of viral mRNAs and has been shown to persist in hepatocytes even with successful cellular and humoral control of the infection indicated by HBsAg/anti-HBs seroconversion. With the impairment of host defense systems, cccDNA can evade host immunity and actively replicate again [19]. The majority of healthy individuals, positive for anti-HBc, which had been assumed to denote a past history of transient HBV infection, were latently infected with the episomal form of HBV accompanied by ongoing viral replication and few nucleotide mutations in the precore and core regions [20].

## 4. Clinical Impact of OBI

OBI is a complex entity that comprises many conditions and different situations [20]. OBI may be involved in several clinical contexts as follows: reactivation of the infection and consequent development of the HBV- related liver disease; transmission of the “occult” virus mainly through blood transfusion and orthotopic liver transplantation (OLT) with consequent hepatitis B in the recipient; the effect on occurrence and progression of the CLD; and the role in hepatocarcinogenesis [21]. 

In OBI, HBV reactivation can be induced by treatment of cancers and autoimmune diseases [22, 23]. Development of a classic hepatitis B that often has a severe clinical course is possible if suppression of viral replication discontinued as in several conditions including HIV infection, hematological malignancies, patients undergoing chemotherapy, transplantation (bone marrow, liver, or kidney), and treatment with potent immunosuppressive drugs like rituximab (anti-CD20), alemtuzumab (anti-CD52), or infliximab (antitumor necrosis factor) [20].

Different reports suggest that OBI could be responsible for the acceleration of chronic hepatitis C virus (HCV) progression and interfere with treatment response [24, 25].

OBI is found in up to 30% of serum samples and 50% of liver biopsies of patients with chronic hepatitis C and 20% and 30% in subjects with cryptogenic liver disease [26, 27]. 

OBI may favor or accelerate the progression toward cirrhosis, associated with the most severe forms of liver disease in HCV-infected patients. Preliminary evidence suggests a possible involvement in faster progression of posttransplant liver disease in HCV-positive patients with OBI in donor or recipient [28].

HCV has been suspected to strongly suppress HBV replication up to the point where it determines OBI development in coinfected individuals. However, more recent studies have brought into question the interplay between HCV and HBV and when the in vitro cotransfection experiments were conducted, no interference between the two viruses was noted [29].

OBI is the major cause of posttransfusion hepatitis B in western countries and in countries like India and Taiwan, with higher risk of transmission than for HCV or HIV [30–32].

Hepatitis B after OLT with OBI in donor is a well-known cause of de novo hepatitis in the HBV-negative recipient but appears to have very low rates of occurrence in cases of kidney, heart, and bone marrow transplantation as a consequence of the fact that hepatocyte is a reservoir of HBV cccDNA [11]. 

Anti-HBV prophylaxis (with hepatitis B immunoglobulin, lamivudine, or their combination) appears to be very effective in preventing de novo HBV hepatitis in the recipients but not to avoid HBV reinfection [33].

Recently, a meta-analysis showed an increased risk of HCC associated with OBI with an odds ratio of 2.9 (95% CI: 1.6–4.1) in retrospective and prospective studies [34]. It is generally believed that OBI maintains most of the pro-oncogenic properties and can contribute to hepatocellular transformation through the same direct and indirect mechanisms that subtend HCC development in overt HBV infection [21]. 

 Aflatoxin B 1 has synergistic hepatocarcinogenesis with chronic HBV infection. Aflatoxin B 1 exposure is common in the area of high prevalence for chronic HBV infection. This effect increased the risk of HCC more than 8-fold compared to HBV infection alone [35]. OBI must be investigated in the following clinical situations: (1) solid organ, hematopoietic stem cell transplantation, and blood transfusion; (2) cryptogenic chronic hepatitis and hepatocellular carcinoma unrelated to HCV, atypical alcoholic hepatitis; (3) immunosuppresive therapy; (4) haemodialysis; (5) chronic hepatitis C especially those with flare in liver enzymes [36]. 

Consequently, it is important to detect high-risk groups for occult HBV infection [16, 37–39].

Clinicians must be aware of these clinical events and establish a standard strategy to prevent HBV reactivation [40].

## 5. Epidemiology 

OBI was reported for the first time more than 30 years ago in the context of blood transfusion of anti-HBc-positive donor as the only marker of HBV infection [41]. No standardized assays evaluating the analysis of occult HBV on liver specimens [16]. Thus OBI prevalence is generally underestimated [21].

Prevalence in different areas and individuals categories, with lack of standardization of laboratory techniques and differences in selection criteria of subjects, does not allow meaningful comparisons [11]. 

However, OBI prevalence seems to be higher among subjects at high risk for HBV infection and with liver disease than among individuals at low risk of infection and without liver disease [42]. 

The prevalence of OBI is estimated to be 4–25% in anti-HBc-positive patients [43].

OBI is more prevalent in certain groups such as HCV and HIV populations. Many studies have found a higher OBI prevalence in subjects with than those without chronic liver disease. There is a wide variation in prevalence of OBI in different case series in patients with cryptogenic chronic hepatitis, HCC, and in HCV-or HIV-infected individuals [44].

This variability depends on the difference in endemicity of HBV infection, utilized assays in the studies, and studied populations in different parts of the world [11].

## 6. HBV Reactivation

Two decades ego, OBI and resolved or past hepatitis B were not recognized to be at risk of HBV reactivation when receiving conventional systemic chemotherapy [45]. But now patients with OBI represent an important group with a high risk for reactivation, especially in endemic areas in the same way as HBsAg-positive patients [46]. Definition of HBV reactivation among patients with OBI has been reported as the reappearance of HBsAg or HBV-DNA in the blood [47]. With resolved hepatitis, HBV reactivation usually begins later than 4 months [48].

Because of the low risk of HBV reactivation in patients with resolved infection with anti-HBs titer >10 IU/L, close followup of LFTs was thought to be sufficient in immunosuppressive treatment groups [49, 50]; although serial HBV-DNA monitoring is a reasonable strategy.

## 7. Risk Factor of HBV Reactivation

Certain host and viral factors are associated with the risk of HBV reactivation in patients with OBI. Host factors include intensity of immunosuppression especially with rituximab plus steroid and hematopoietic stem cell transplantation, and viral factors include absence of anti-HBs before chemotherapy, decrease in anti-HBs during chemotherapy, detectable HBV DNA in the serum, HBV genotype B, and mutations in the precore and core promoters [51, 52].

The degree of immunosuppression in the frequency and severity of HBV reactivation is highlighted by reports of severe reactivation following aggressive forms of chemotherapy or immune suppression like in lymphoma than in those with solid tumors [53].

This can be attributed to the fact that hematological disease itself induces a greater degree of immunosuppression or that chemotherapy is stronger in cases of hematological malignancies [54].

Several reports highlight the increasing incidence of reverse seroconversion in anti-HBs-positive patients after chemotherapy regiments containing anti-CD20 (rituximab) and or autologous hematopoietic stem cell transplantation for hematological malignancies ++ with diverse outcomes [51, 55–58].

High percentage of subclinical reactivation of HBV in HBsAg negative after-solid organ transplant, without clinical hepatitis reported [59, 60].

The risk of occult HBV transmission from donors who are HBsAg-negative and anti- HBc-positive is very low after kidney, heart, or bone marrow but higher (17–90%) in orthotopic liver transplantation especially if the recipient is negative for all HBV serum markers [41, 61]. 

HBV-related hepatitis following solid organ transplantation from anti-HBc positive donors to healthy recipient usually has a benign course and is often less severe when compared to hepatitis B that develops as a result of HBV reactivation in anti-HBc positive recipient [40].

Persistence of the virus in the hepatocyte nuclei as cccDNA in those with serological evidence of resolved infection is considered mostly to be important risk factor in patients with HBV reactivation following systemic chemotherapy [3].

Quantification of HBV core-related antigen (HBcrAg) which has been reported to be correlated with the amount of cccDNA in the liver would be expected to represent a predictive marker for HBV reactivation [62].

Different non-A genotypes (especially B genotypes) and precore and core promoter mutations, which are prevalent in B and C genotypes of Asians, have been reported to be associated with the reactivation of HBV in the setting of systemic chemotherapy [63].

Reactivation of the patient's HBV infection differs according to the replication status prior to systemic chemotherapy as well as to the degree of immunosuppression. The risk of HBV reactivation in the HBsAg-negative patients was 2.7% versus 48% in HBsAg-positive patients [64]. 

According to recommendations neither patients with serological evidence of resolved HBV infection needed prechemotherapy assessment of viral load, nor any of them were candidates for preemptive nucleoside analogue treatment [49].

## 8. Preventive Measure

At least one-third of patients die from HBV reactivation despite treatment with lamivudine. It would probably be best to administer preventive treatment for HBV reactivation as this would inhibit the development of hepatitis and mortality with the aim of inhibiting the replication of HBV [65].

In OBI, exact risk of HBV reactivation depends on viral and host factors but associations with HBV reactivation have not been analyzed thoroughly [66].

Initiating antiviral treatment after hepatitis onset may be insufficient to control HBV reactivation and fulminant hepatitis and mortality following HBV reactivation is higher than acute hepatitis B [66].

Antiviral prophylaxis should be continued for ≥6 months after stopping chemotherapy and for certain immunosuppressive therapies, such as rituximab; it may be better to maintain the prophylaxis until restoration of host immunity [67]. There is insufficient evidence to recommend routine prophylaxis, but treatment is recommended for patients with risk factors and in other cases, followup and early treatment should be recommended in case of reactivation [65]. All patients who receive chemotherapy and immunotherapy should be tested for serologic markers of HBV infection, including HBsAg, anti-HBc, and anti-HBs before any chemotherapy or immunosuppressive therapy, and monitored for several months or years after stopping treatment because antibody titers may be reduced by the immunosuppressive therapy [40]. Antiviral drugs should be initiated to OBI, especially in the absence of anti-HBs which are potentially at greater risk for HBV reactivation prior to chemotherapy and continued for ≥6 months after stopping immunosuppressive treatment [68, 69]. The appearances of HBsAg and HBV DNA in up to 50% of anti-HBc positive patients undergoing bone marrow transplantation have been reported. The serial determination of anti-HBs in the serum of these bone marrow recipients has shown a steady decline to undetectable levels by 1–3 years after transplantation. Decreased titers of anti-HBs have been reported to be closely associated with HBV reactivation and with the loss of anti-HBs, HBV DNA increases and HBsAg reappears [70, 71]. Prophylaxis with antiviral agents prevents reactivation of OBI in most of transplant cases with HBsAg-negative and anti-HBc-positive donors and it is not known if prior hepatitis B immunization with an optimal anti-HBs response can modulate or abort the infection [40].

Current data are insufficient to recommend routine prophylaxis and antiviral therapy to prevent HBV reactivation for HBVDNA and HBsAg-negative but anti-HBc and/or anti-HBs positive patients, except in intense chemotherapy like rituximab. Thus early identification of virologic reactivation is essential to start antiviral therapy and prevent the occurrence of hepatitis B [19, 51, 65]. Serial HBV-DNA monitoring (monthly during and after chemotherapy for at least 1 year) is a reasonable strategy recommended by the latest Japanese guidelines; in this regard multicenter clinical trial in Japan is now continued [72]. With HBV-DNA NAT antiviral therapy begins when the result is >30 IU/mL and with a highly sensitive HBsAg assay (low limit of detection <0.1 ng/mL) antiviral therapy begin when the test becomes positive [20]. Although in blood transfusion the risk of transmission is insignificant when anti-HBs is present in the blood, caution is recommended when immunodeficient patients receive anti-HBc-positive and anti-HBs-positive donations [40]. The use of HBV-DNA NAT and multivalent anti-HBs antibodies in the HBsAg assays is recommended for detection of true and false OBI, respectively, and to minimize the risk of HBV transmission through transfusion [40]. This is important because almost 50% of transfused blood in Western Europe is given to immunodeficient patients [73].

## 9. Conclusions

For detection of OBI -HBV DNA nucleic acid testing should be implemented even if anti-HBc and anti-HBs were negative especially in endemic area and in suspected high-risk cases (populations at high risk of parenterally transmitted infections) with probable previous exposure before blood and organ donation, transplantation, and chemotherapy and in hemodialysis and cryptogenic chronic hepatitis. The use of multivalent anti-HBs antibodies in the HBsAg detection kits is strongly recommended. All patients receiving chemo- and immunotherapy should be tested at least once for anti-HBc antibodies before starting therapy and monitored periodically for ALT elevations. If ALT elevations occurred, the diagnosis of HBV reactivation must be established with further testing before initiation of antiviral prophylaxis. Optimal duration of prophylaxis in different risk populations should be clarified or even individualized in the future.
